# Effects of irrigation and organic fertilizer on pumpkin yield, quality, and water-fertilizer use efficiency in arid northwest China

**DOI:** 10.3389/fpls.2025.1517761

**Published:** 2025-02-07

**Authors:** Meng Yin, Jinxia Zhang, Liangliang Du, Lin Ding, Tao Zhong, Pengliang Tian, Runheng Yang

**Affiliations:** ^1^ College of Water Conservancy and Hydropower Engineering, Gansu Agricultural University, Lanzhou, China; ^2^ Rural Water Conservancy Institute, Gansu Academy for Water Conservancy, Lanzhou, China

**Keywords:** water-fertilizer coupling, pumpkin yield and quality, irrigation water use efficiency, partial fertilizer productivity, TOPSIS

## Abstract

Due to the increasing water scarcity and the need for sustainable agricultural practices in arid regions, optimizing water and fertilizer management is crucial for enhancing crop productivity and resource efficiency. Field experiments in 2022 and 2023 in northwestern China’s arid region explored the impacts of irrigation volume, organic fertilizer use, and their coupling on pumpkin yield, quality, and water-fertilizer efficiency. The study included ten treatments with a completely randomized two-factor design, comprising three irrigation quotas, three organic fertilizer application rates and a control group (CK). The results showed that the organic fertilizer application significantly enhanced soil moisture content, which peaked at a depth of 50 cm. Irrigation quota and organic fertilizer application had a highly significant impact on pumpkin vine length and stem diameter (P < 0.01), with a significant interaction between the two factors (P < 0.05). The rate of dry matter accumulation in pumpkin peaked at 60 ~ 80 days after sowing, with a trend of F2 > F3 > F1 in dry matter accumulation at identical irrigation quota. The effects of irrigation volume, organic fertilizer application and water-fertilizer coupling on pumpkin yield, irrigation water use efficiency (IWUE), partial fertilizer productivity (PFP) and pumpkin quality were statistically highly significant (P < 0.01). Specifically, increasing the irrigation volume from W1 to W3 increased the yield by 17.36%. However, pumpkin yield initially increased and then decreased in response to increasing organic fertilizer application. IWUE increased and then decreased with the increase of organic fertilizer application, while PFP increased with the increase of irrigation volume. Regression analysis revealed that the optimal range for irrigation quota to ensure pumpkin quality was 430 ~ 506 m^3^·ha^-1^, and that for organic fertilizer application was 5,373 ~ 6,570 kg·ha^-1^. When only quality indicators were considered, the W2F2 treatment performed well. However, from the comprehensive evaluation of pumpkin yield, quality, and water and fertilizer use efficiency using the TOPSIS method, the W3F2 treatment was identified as the most suitable among the water- fertilizer coupling management modes considered in this study for pumpkin cultivation in the arid northwestern China.

## 1 Introduction

Pumpkin is a rich source of dietary fiber, making it suitable for various food and health products. Moreover, its cultivation by farmers as one of the most common cash crops not only brought high economic benefits but also contributed to its widespread availability on the market (Bai et al., 2020). According to FAO statistics, the global pumpkin production reached 2.38 million tonnes in 2021, of which China accounted for 31.2% with 0.74 million tonnes (Food and Agricultural Organization, 2021). The Northwest Arid Region is the main production area of pumpkin production in China. However, the Northwest Arid Region is located deep in the interior and is characterized by low rainfall and a dry climate. The average annual rainfall is less than 200 mm, while the annual evaporation is more than 1000 mm (Shan et al., 2020; Wu and Du, 2020). Pumpkin is highly drought tolerant and does not have strict soil requirements (Liu et al., 2023), making it valuable and ecologically adaptable for cultivation in this area.

In recent years, the substitution of chemical fertilizers with organic fertilizers has received increasing attention as a sustainable approach for agricultural development, by improving soil physical, chemical, and biological properties (Tang et al., 2019; Wang et al., 2018). The use of organic fertilizer could enhance soil ecological environment, improve soil fertility, activate soil nutrients, raise nutrient absorption and utilization by crops, promote crop growth and yield increase, and enhance crop quality (Wu et al., 2024; Zhao et al., 2024). The problems of uneven distribution of water resources, irrational irrigation practices (Cai et al., 2022), excessive nitrogen fertilizer application, declining water and fertilizer efficiency (Qi et al., 2020), farmland ecological pollution (Luo et al., 2024), poor soil quality (Hui et al., 2022), and low quality of agricultural products in the arid region of northwestern China had greatly hindered the yield and quality of pumpkin, and prevented the sustainable development of the economy and society in this region (Chen et al., 2019a; Sá et al., 2023). Pumpkin yield and quality were significantly influenced by the use of irrigation levels and fertilization practices (Zhong et al., 2024). Insufficient soil moisture restricts pumpkin growth, resulting in reduced yield and poor quality (Li et al., 2022). Chen et al. (2019b) observed a nonlinear relationship between fertilizer application and pumpkin yield, with an initial increase followed by a subsequent decrease.

Water plays a key role in enhancing the effectiveness of fertilizer, while fertilizer is the key to unlocking the productive efficiency of the soil-water system. Therefore, the determination of the appropriate amount of fertilizer must be closely linked to the water status (Li et al., 2023; Stoleru et al., 2020). Coupled with the lack of scientific and effective irrigation and fertilizer management measures by local farmers, the application amount of irrigation water and fertilizer to pumpkin far exceeded its actual needs (Zhang et al., 2023a), resulting in a waste of valuable resources and a reduction in their use efficiency. Meanwhile, excessive irrigation and fertilization could aggravate soil salinization (Zhang et al., 2023b) and lead to soil contamination in farmland (Zeng et al., 2019), severely hindering the growth and development of pumpkin. Exceeding a certain range of irrigation and fertilization application could lead to a decrease in pumpkin yield, quality and water and fertilizer use efficiency (Wang et al., 2021; Yu et al., 2020). Reasonable irrigation and fertilization practices have been shown to improve pumpkin yield, quality, water use efficiency, and fertilizer partial productivity (Ali et al., 2018; Wang and Xing, 2017). Liu et al. (2019) revealed the nonlinear impacts of irrigation and fertilization on both crop yield and quality. The findings showed that the optimized crop performance could be achieved with moderate water and fertilizer inputs, while excessive inputs resulted in reduced yield, compromised quality, and reduced efficiency of water and fertilizer use. Therefore, it would be crucial to ensure a proper application of water and fertilizer to maximize pumpkin yield, improve its quality and increase the use efficiency of water and fertilizer.

Although irrigation and organic fertilizer application have been widely used in pumpkin production in the arid regions of northwest China, previous studies have mainly focused on individual factors (Li et al., 2022; Walters, 2020; Yavuz et al., 2015) and water-nitrogen coupling (Li et al., 2019; Su et al., 2023). The comprehensive effects of irrigation amount, organic fertilizer application rate, and their interaction on pumpkin yield, quality, as well as water and fertilizer use efficiency remain unclear. Therefore, a study would be carried out aiming to the impacts of irrigation amount and organic fertilizer application on soil water content, pumpkin growth, dry matter accumulation, yield, quality, water use efficiency, and fertilizer partial productivity. Regression analysis would also be used to identify the optimum intervals of irrigation quota and organic fertilizer application to ensure the optimal pumpkin quality. Based on TOPSIS, pumpkin yield, quality, and use efficiency of water and fertilizer would be comprehensively evaluated. Eventually, a coupling scheme of irrigation amount and organic fertilizer application suitable for pumpkin cultivation in the arid region of Northwest China would be proposed. The results of this research would provide a theoretical basis and scientific guidance for enhancing the yield and quality of pumpkin while ensuring the efficient use of water and fertilizer resources in the region.

## 2 Materials and methods

### 2.1 Overview of test area

**Figure 1:**
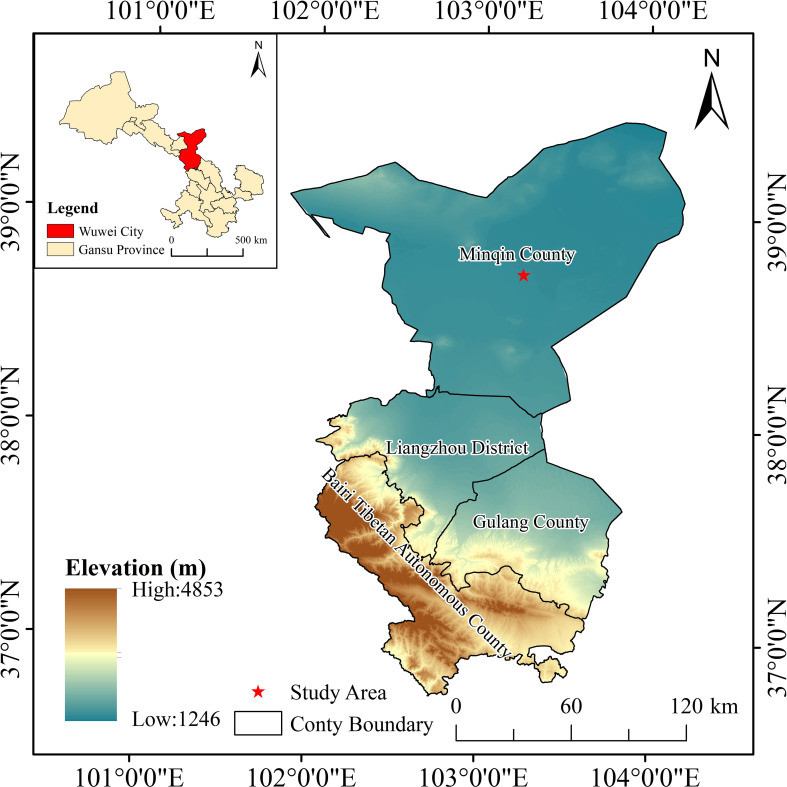
Geographical location of the test area (located in Minqin County, Gansu Province, China), indicated by the coordinates 38°37’ N latitude and 103°05’ E longitude, and an elevation of 1300 meters above sea level.

**Figure 2:**
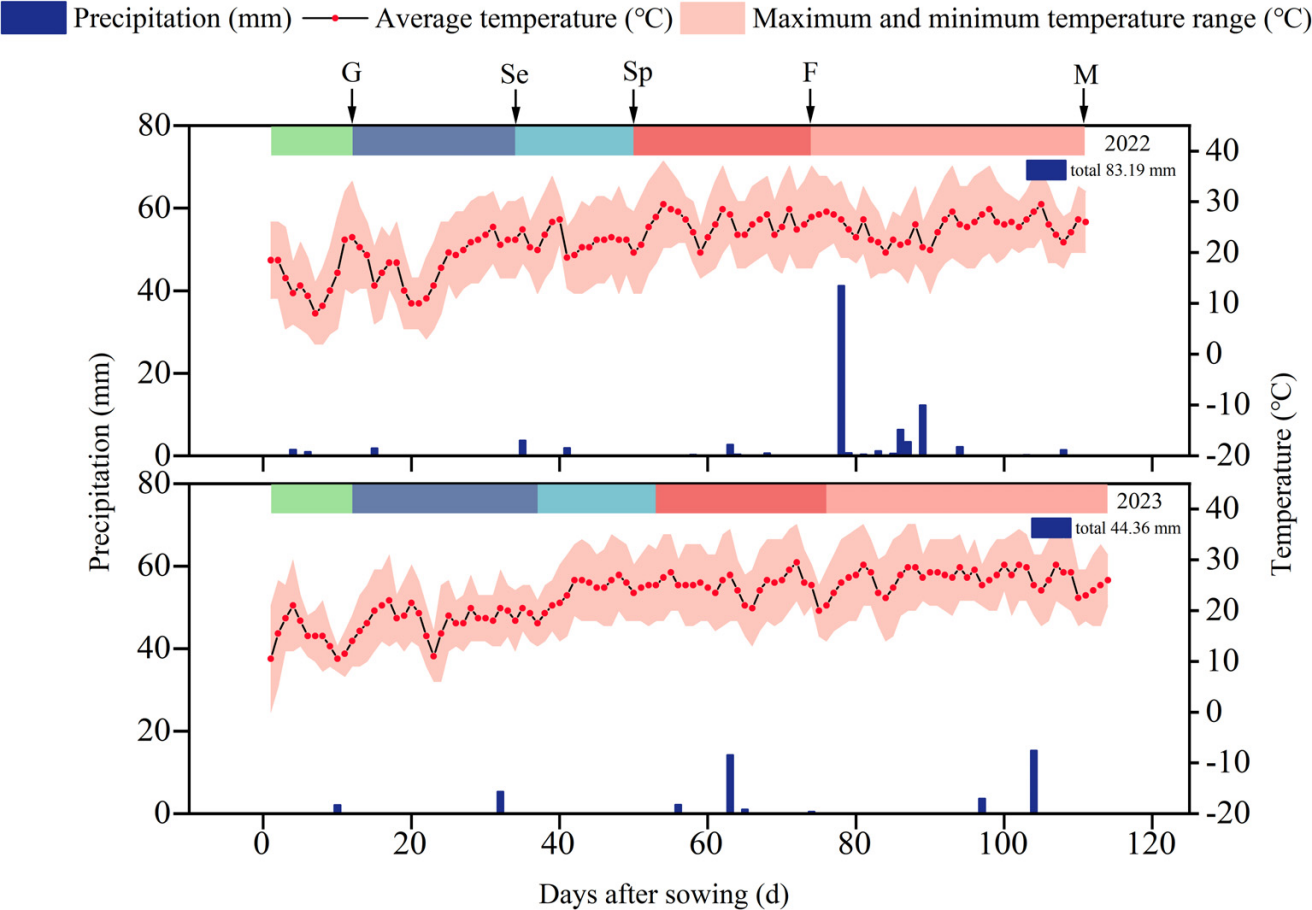
Daily precipitation and average air temperature during pumpkin growing season in 2022 and 2023. Different color blocks indicate different growth stages of pumpkin. G is germination stage, Se is seedling stage, Sp is the sprouting stage, F is flowering stage, M is maturity stage.

Field experiments were conducted at the Minqin Irrigation Experimental Station of Gansu Academy of Water Conservancy Science from April to September in 2022 and 2023. The experimental station is located in Dongda Village (38°37’ N, 130°05’ E, altitude 1300 m), Datan Township, about 13.5 km north of Minqin County. The geographical location of the experimental area is shown in **Figure 1**. This area represents a typical continental desert climate at the junction of an oasis and the Tengger Desert. The average annual temperature is 7.8°C, with an average annual precipitation of 110mm and an average annual evaporation of 2644 mm. **Figure 2** illustrates the precipitation and average temperature during the test period in both years. The total precipitation during the pumpkin growing season in 2022 and 2023 is 83.19 mm and 44.36 mm, respectively. The soil within the experimental site consists of clay loam from 0 to 60 cm, gradually transitioning into sandy loam below this layer. The average dry bulk density of the 0 ~ 100 cm soil was 1.54 g·cm^-3^, the average specific gravity was 2.61 g·cm^-3^, the average porosity was 42.80%, the average field water holding capacity was 23.00% and the permanent wilting point was 7.65%. The soil organic matter content of the trial area was 0.53%, total nitrogen 0.045%, total phosphorus 0.12%, total potassium 1.67%, alkaline dissolved nitrogen 18.7 mg·kg^-1^, quick release phosphorus 15.98 mg·kg^-1^, quick release potassium 155 mg·kg^-1^, and pH 7.96.

### 2.2 Experimental design

The experiment employed a completely randomized two-factor design, with two control factors: irrigation quota and organic fertilizer application amount. The irrigation quota and organic fertilizer application amount were selected based on local agricultural practices in arid northwest China. The irrigation quota consisted of three levels: W1 (375 m^3^·ha^-1^), W2 (450 m^3^·ha^-1^), and W3 (525 m^3^·ha^-1^). And there were three levels of organic fertilizer application: F1 (4500 kg·ha^-1^), F2 (5700 kg·ha^-1^), and F3 (6900 kg·ha^-1^). The control treatment (CK) represented the level of conventional irrigation and chemical fertilizer application used by local farmers, with an irrigation quota of 525 m^3^·ha^-1^ and a fertilizer level consisting of a base fertilizer of 300 kg·ha^-1^ diammonium phosphate and 450 kg·ha^-1^ urea, along with two top dressings of 300 kg·ha^-1^ urea each. A total of ten treatments were applied, with each treatment replicated three times on thirty 75 m^2^ (30 m×2.5 m) plots. **Table 1** provides detailed information on the irrigation and fertilization scheme used in this study. Notably, top dressing was applied twice during both the late sprouting and flowering stages. To ensure seedling emergence, all treatments received one a pre-sowing irrigation event at a rate of 450 m^3^·ha^-1^. Throughout the growing season, irrigation was conducted thrice according to the experimental design.

**Table 1:** Experimental design scheme.

Treatments	Base Fertilizer (kg·ha^-1^)	Top dressing fertilizer (kg·ha^-1^)	Top dressing times	Irrigation quota (m^3^·ha^-1^)	Irrigation times
F1W1	3900 (Solid organic fertilizer)	300 (Liquid Organic Fertilizer)	2	375	3
F1W2	3900 (Solid organic fertilizer)	300 (Liquid Organic Fertilizer)	2	450	3
F1W3	3900 (Solid organic fertilizer)	300 (Liquid Organic Fertilizer)	2	525	3
F2W1	4800 (Solid organic fertilizer)	450 (Liquid Organic Fertilizer)	2	375	3
F2W2	4800 (Solid organic fertilizer)	450 (Liquid Organic Fertilizer)	2	450	3
F2W3	4800 (Solid organic fertilizer)	450 (Liquid Organic Fertilizer)	2	525	3
F3W1	5700 (Solid organic fertilizer)	600 (Liquid Organic Fertilizer)	2	375	3
F3W2	5700 (Solid organic fertilizer)	600 (Liquid Organic Fertilizer)	2	450	3
F3W3	5700 (Solid organic fertilizer)	600 (Liquid Organic Fertilizer)	2	525	3
CK	300 (diammonium phosphate)、450 (urea CO(NH_2_)_2_)	300 (urea CO(NH_2_)_2_)	2	525	3

**Figure 3:**
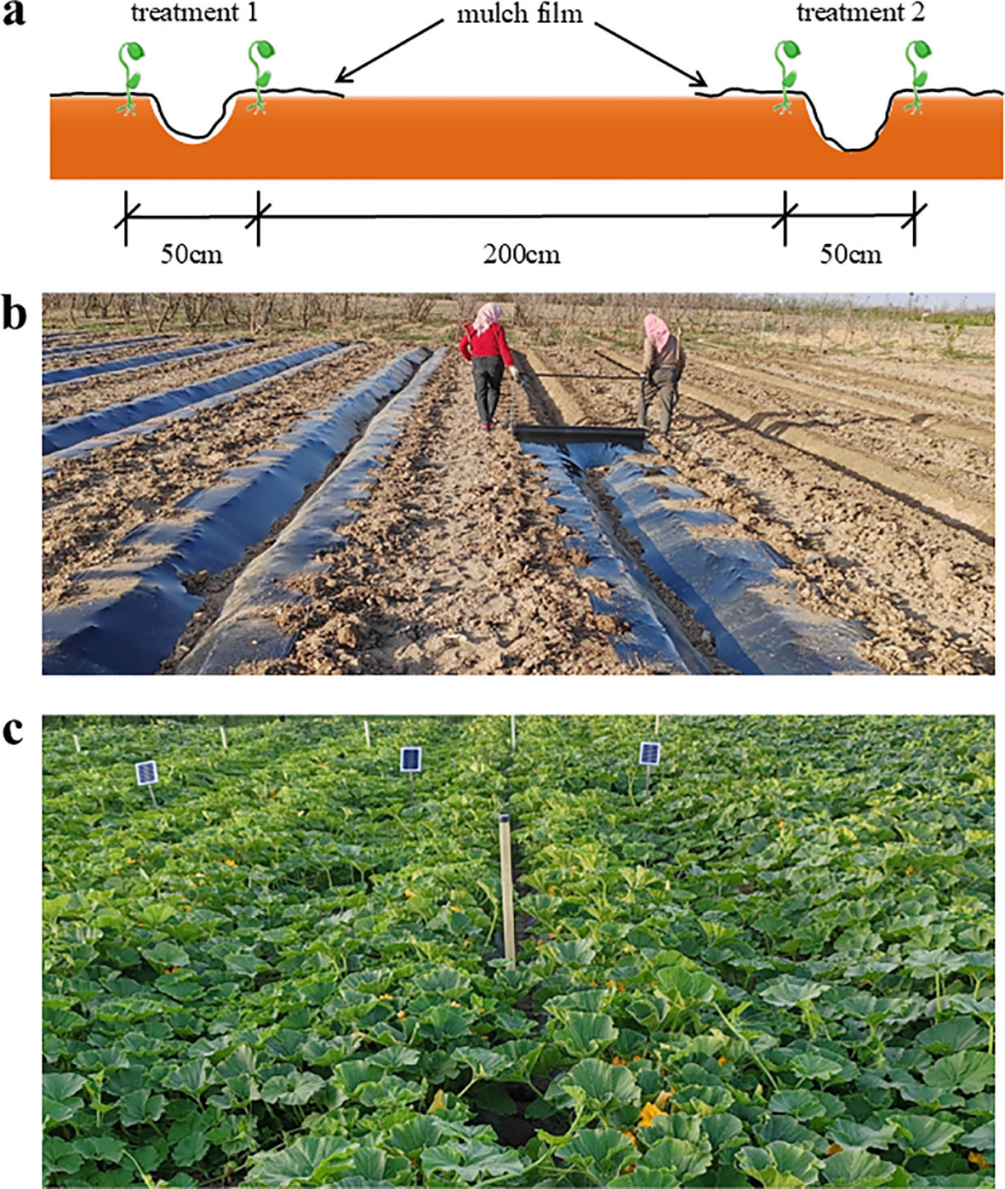
The design of field experiment in 2022 and 2023. Planting pattern **(A)**. Photos of pumpkin planting photo **(B)** and pre-harvest pumpkin photo **(C)**. Photos **(B, C)** were taken by the authors.

The experimental pumpkin variety, ‘Sweet Pumpkin’, was sown on 24 April 2022 and 29 April 2023, and harvested on 12 August 2022 and 20 August 2023, respectively. The experiment employed a single furrow planting pattern with one film covering two rows. The large row spacing was set at 200 cm, while the small row spacing was maintained at 50 cm with a planting distance of 30 cm. The planting pattern is shown in **Figure 3**. Prior to sowing, the land was raked, leveled, furrowed and irrigated. The organic fertilizer used in this study was sourced from Lanzhou Xindali Water and Fertilizer Integrated Service Co., Ltd., and met the following criteria: N + P_2_O_5_ + K_2_O ≥18%, effective bacteria (Bacillus subtilis + Bacillus licheniformis) ≥0.5 million/ml, amino acids ≥3%.

### 2.3 Elements and methods of determination

#### 2.3.1 Soil moisture content

At each growth stage of pumpkin, soil samples were taken at intervals of 20 cm between 0 and 100 cm depth using the soil drilling method, and then analyzed for moisture content by a drying technique.

#### 2.3.2 Vine length and stem diameter

At each growth stage of pumpkin, three representative plants with uniform growth characteristics were randomly selected from each plot based on visual inspection and labeled. The vine length and stem diameter were measured using a tape and a caliper, respectively.

#### 2.3.3 Dry matter weight

At each growth stage of pumpkin, three plants that appeared to be of average growth and health were randomly selected from each plot based on visual assessment and subjected to oven drying at 105°C for 30 minutes, followed by further drying at 75°C until a constant weight was reached. The dry matter weight of the above-ground portion in pumpkin plant was measured using an electronic balance with an accuracy of 0.01 g.

#### 2.3.4 Pumpkin yield

At maturity, three representative plants that exhibited average fruit size and uniformity were randomly sampled from each plot based on visual inspection to quantify the pumpkin fruit yield.

#### 2.3.5 Irrigation water use efficiency and partial fertilizer productivity

The irrigation water use efficiency (IWUE, kg·m^-3^) is calculated according to Equation 1:


(1) IWUE=Y/I

Where ***Y*** is the yield of pumpkin, kg·ha^-1^; ***I*** is the irrigation amount, m^3^·ha^-1^.

The calculation of the partial fertilizer productivity (PFP, kg·kg^-1^) is shown in Equation 2:


(2) PFP=Y/F

Where ***Y*** is the yield of pumpkin, kg·ha^-1^; ***F*** is the total amount of fertilizer, kg·ha^-1^.

#### 2.3.6 Determining pumpkin quality

After the pumpkin reached maturity, three pumpkins that appeared to be of average size and uniformity were selected from each plot based on visual inspection to determine the levels of various quality indices. The concentrations of soluble sugars, vitamin C, and soluble solids in the pumpkin samples were quantified using a kit supplied by Beijing Box Biotechnology Co., Ltd.

### 2.4 Data analysis

Data compilation was done using Excel 2021, drawing was conducted with Origin 2023 software. Statistical analysis was performed using SPSS 26.0 software, with one-way ANOVA for each indicator among different treatments and Duncan’s method for multiple comparisons (P<0.05). Two-way ANOVA was employed to test the effects of irrigation quota and organic fertilizer application, as well as their interaction on the pumpkin (P<0.05). The different coupling modes of irrigation quota and organic fertilizer application were comprehensively evaluated using the TOPSIS method (Yu et al., 2023).

## 3 Results and analysis

### 3.1 Effect of water and fertilizer coupling on soil moisture content

**Figure 4:**
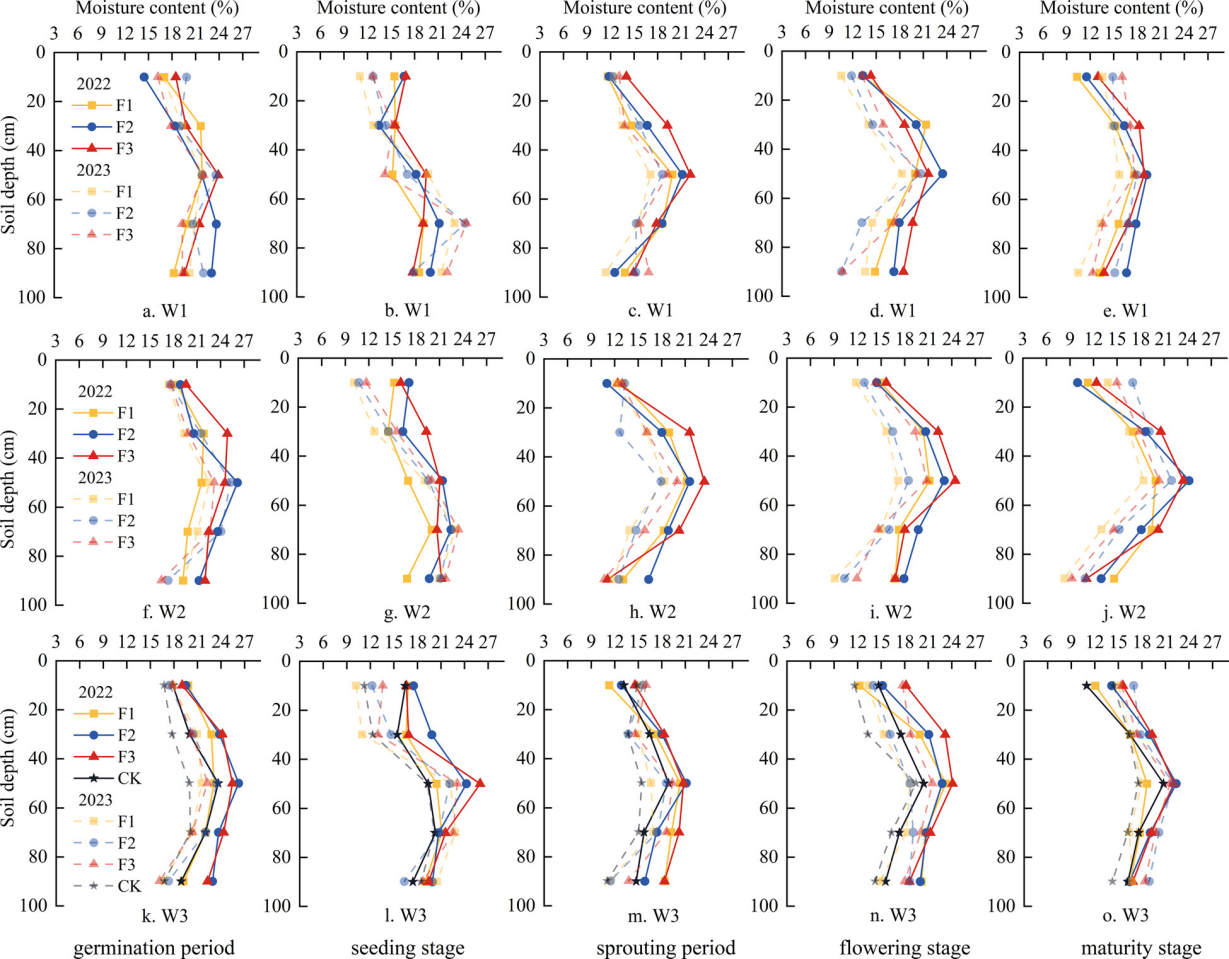
Effects of different water and fertilizer regulation on the water content of 0 ~ 100cm soil layer in each growth period of pumpkin. The actual line is the soil moisture content of each growth period in 2022, and the imaginary line is the soil moisture content of each growth period in 2023.

The dynamic changes of soil moisture content within the 0 ~ 100 cm depth during pumpkin growth period under different water and fertilizer coupling are illustrated in **Figure 4**. The temporal trend of soil moisture content within the 0 ~ 100 cm soil layer for each treatment remained generally consistent from 2022 to 2023. In terms of spatial distribution, there was an initial increase followed by a decrease in soil moisture content with increasing depth. Specifically, the soil moisture content gradually increased in the uppermost 0 ~ 50 cm soil layer, while it decreased with further depth in the lowermost 50 ~ 100 cm soil layer. Temporally, there was a decline in soil moisture content across all layers as the growth period progressed.

From an interannual perspective, at constant irrigation quotas, the soil moisture content at different growth stages increased with improving the application of organic fertilizer. Compared to the control group (CK), the average soil moisture content in the 100 cm soil layer increased by 4.3% ~ 12.48% for F1, 7.8% ~ 21.7% for F2, and 11.64% ~ 28.29% for F3 treatments, respectively. At the constant organic fertilizer application level, increasing irrigation amounts also resulted in higher soil moisture contents. The average soil moisture content in the 100 cm soil layer increased by 3.21% ~ 11.74%, 6.86% ~ 15.73% and 9.67% ~ 28.28% for W1, W2 and W3 treatments respectively compared with CK. These findings demonstrate that the coupling of water and fertilizer has a positive impact on the improvement of soil moisture content. Thus, applying organic fertilizer and increasing irrigation quotas are beneficial to improve soil moisture content.

### 3.2 Effects of water and fertilizer coupling on vine length and stem diameter of pumpkin

**Figure 5:**
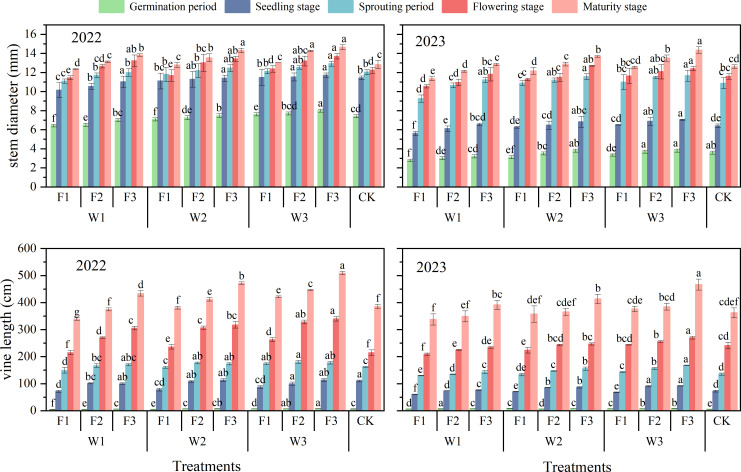
Effects of water and fertilizer coupling on stem diameter and vine length of pumpkin. Different lower case letters indicate significant differences between different treatments in the same growth period (P<0.05).

The effects of irrigation quotas and organic fertilizer application rates on pumpkin vine length and stem diameter were highly significant (P < 0.01), while the interaction between water and fertilizer significantly influenced both vine length and stem diameter (P < 0.05) (**Figure 5**). In particular, in both years, stem diameter at the F3 level was significantly greater than that at the F1 and F2 levels (P < 0.05). Compared to the control (CK), stem diameter of pumpkin in the W1F3, W2F3, and W3F3 treatments increased by 7.72%, 11.5%, and 14.3%, respectively in 2022. In 2023, these increases were 1.78%, 8.53%, and 13.77%, respectively. With the exception of the F2, vine length during the sprouting period of pumpkin in 2022, which was significantly greater than that of F3 under W2 conditions (P < 0.05), vine length at the F3 level was significantly greater than that at F1 and F2 levels under W1, W2, and W3 conditions in all growth periods of both 2022 and 2023 (P < 0.05). At maturity stage, compared to CK, pumpkin vine lengths increased by 12.49%, 22.27%, and 31.97% respectively in W1F3, W2F3, and W3F3 treatments in 2022. Similarly, increases of 8.10%, 14.18%, and 28.82% were observed in these treatments during the study year in 2023 as well. These findings indicated that adequate irrigation coupled with organic fertilizer application enhanced both stem diameter growth and vine length development in pumpkin.

### 3.3 Effect of water and fertilizer coupling on pumpkin dry matter

**Figure 6:**
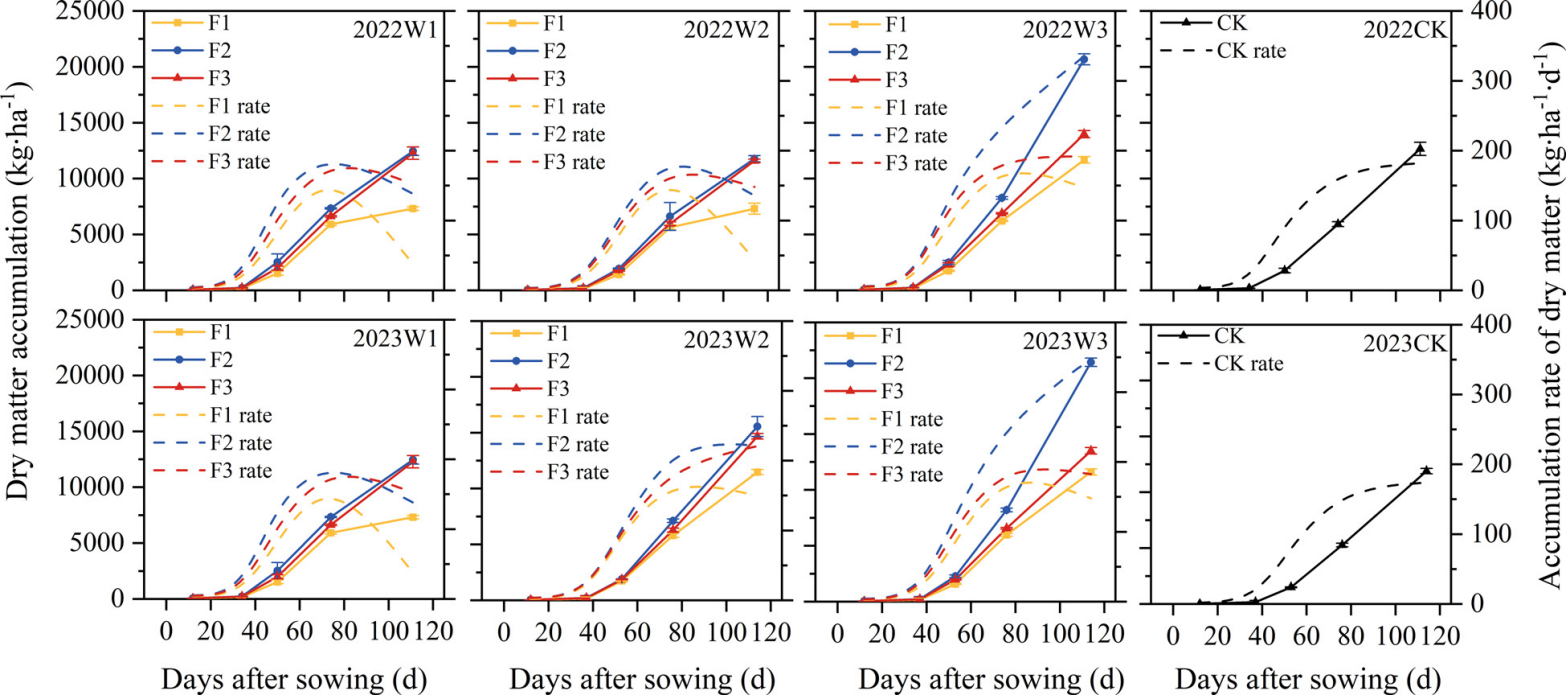
Effects of water and fertilizer coupling on dry matter accumulation and dry matter accumulation rate of pumpkin. The solid line and the dotted line represent dry matter accumulation and dry matter accumulation rate, respectively.

The above ground dry matter accumulation of pumpkin exhibited a curvilinear pattern throughout the entire growth period (**Figure 6**): increases were slow in both the early and late stages, with the most rapid growth occurring in the middle stage, and the maximum dry matter accumulation of pumpkin decreased with decreasing irrigation amounts. At identical fertilization levels, the two-year average maximum dry matter accumulation for W2 and W3 treatments increased by 33.2% and 73.8% respectively compared to W1. Within each growth period under consistent irrigation quotas, dry matter accumulation followed a trend of F2 > F3 > F1 for the different fertilization treatments. Notably, the average dry matter accumulation for W3F2 in each growth period was higher than that of the CK treatment by 80.9%, 33.5%, 46.3%, 47.1%, and 71.5%, respectively.

### 3.4 Effects of water and fertilizer coupling on pumpkin yield and water-fertilizer use efficiency

**Table 2** revealed that irrigation, organic fertilizer, and water-fertilizer coupling had a highly significant impact on pumpkin yield (P<0.01). As the amount of irrigation increased, there was a significant increase in pumpkin yield. At the same fertilization level, the annual average total pumpkin yield under W3 was 17.36% and 7.91% higher than that of W1 and W2, respectively; and 12.51% higher than that of CK (24689.06 kg·ha^-1^). Among the W1 level, the total yield of W1F3 was the highest in 2022 and 2023, reaching 24635.06 kg·ha^-1^ and 24885.06 kg·ha^-1^, respectively. These values were significantly higher than those of W1F1 by 11.82% and 15.18%, respectively; and 1.7% and 1% higher than those of W1F2, respectively. In comparison to the CK treatment, there was a significant increase in yield for W1F3 in 2022; however, no significant difference was observed between the yields of WIF3 and the CK treatment in 2023. Among the W2 level, the total yield of W2F2 was the highest in 2022 and 2023. These yields were significantly higher than those of W2F1 by 14.24% and 13.4% respectively, and significantly higher than those of W2F3 by 4.98% and 3.95%, respectively. Furthermore, in both years there exhibited a significant increase compared to the CK treatment, exceeding it by 11.91% and 8.91%, respectively. Among the W3 level, the total yield of W3F2 was the highest in 2022 and 2023, exhibiting a significant increase of 23.33% and 18% compared to W3F1, as well as an impressive improvement of 18% and 17% when compared to W3F3. Furthermore, both years were significantly higher than the CK treatment with increases of 27% and 25% respectively. Under identical irrigation conditions (W2, W3) and different fertilization levels, pumpkin yield exhibited an initial increase followed by a subsequent decrease in response to organic fertilizer application.

**Table 2:** Effects of water and fertilizer coupling on pumpkin yield, irrigation water use efficiency and fertilizer partial factor productivity.

2022				2023		
Treatments	Yield (kg·ha^-1^)	IWUE (kg·m^-3^)	PFP (kg·kg^-1^)	Yield (kg·ha^-1^)	IWUE (kg·m^-3^)	PFP (kg·kg^-1^)
W1F1	22030.15 ± 105.41g	13.99 ± 0.07e	4.90 ± 0.02e	21605.14 ± 406.16f	13.72 ± 0.72c	4.80 ± 0.25de
W1F2	24225.80 ± 133.73e	15.38 ± 0.08b	4.25 ± 0.02g	24632.31 ± 353.09de	15.64 ± 0.95ab	4.32 ± 0.26ef
W1F3	24635.06 ± 181.92d	15.64 ± 0.12a	3.57 ± 0.03i	24885.06 ± 181.92d	15.80 ± 0.12a	3.61 ± 0.03g
W2F1	23628.75 ± 183.37f	13.13 ± 0.10f	5.25 ± 0.04d	24131.48 ± 421.46e	13.41 ± 0.75cd	5.36 ± 0.30bcd
W2F2	26992.70 ± 168.01b	15.00 ± 0.09c	4.74 ± 0.03f	27508.70 ± 526.77b	15.42 ± 1.08ab	4.87 ± 0.34cde
W2F3	25713.12 ± 263.11c	14.29 ± 0.15d	3.73 ± 0.04h	26463.12 ± 263.11c	14.70 ± 0.15b	3.84 ± 0.04fg
W3F1	24914.16 ± 131.15d	12.30 ± 0.06h	5.54 ± 0.03b	26705.73 ± 566.08c	13.19 ± 0.71cd	5.93 ± 0.32b
W3F2	30726.13 ± 207.54a	15.17 ± 0.10bc	5.39 ± 0.04c	31476.13 ± 207.54a	15.54 ± 0.10ab	5.52 ± 0.04bc
W3F3	26044.72 ± 167.67c	12.86 ± 0.08g	3.77 ± 0.02h	26794.72 ± 167.67c	13.23 ± 0.08cd	3.88 ± 0.02fg
CK	24119.46 ± 160.08e	11.91 ± 0.08i	17.87 ± 0.12a	25258.67 ± 644.52d	12.47 ± 0.68d	18.71 ± 1.01a
Significance analysis (*F*)						
W	1264.33**	783.26**	581.41**	386.79**	8.16**	15.46**
F	1403.48**	1348.38**	3332.25**	251.68**	31.77**	54.68**
W×F	231.96**	182.60**	100.40**	40.62**	4.29**	1.85ns

Different lowercase letters indicate that there is a significant difference between different treatments at the P=0.05 level, * indicates a significant difference (P<0.05), ** indicates a very significant difference (P<0.01), and ns indicates no significant difference.

The coupling of water and fertilizer in 2022 and 2023 significantly influenced irrigation water use efficiency (IWUE) of pumpkin (P < 0.01). Additionally, the coupling of water and fertilizer in 2022 had a significant impact on partial fertilizer productivity (PFP) (P < 0.01). The effects of irrigation and fertilization on IWUE and PFP were highly significant during both years. IWUE gradually decreased with increasing irrigation amount under the same organic fertilizer level. Conversely, under the same irrigation level (W2, W3), IWUE initially increased and then decreased with increasing organic fertilizer application. Notably, the impact of fertilization on IWUE surpassed that of irrigation. Under the W1F3 treatment, the IWUE of pumpkin in 2022 and 2023 which was significantly higher than that under other treatments. At a constant level of organic fertilizer application, PFP increased with increasing irrigation amount. Conversely, at a fixed irrigation level, PFP decreased with increasing organic fertilizer application. The influence of organic fertilizer application on PFP was found to be more pronounced than that of irrigation. Notably, due to the use of chemical fertilizers in the control treatment (CK), its PFP was significantly greater than that observed in the organic fertilizer treatments. In the W3F1 treatment, the PFP values for pumpkin in 2022 and 2023 were significantly higher than those of the other organic fertilizer treatments.

### 3.5 Effect of water and fertilizer coupling on pumpkin quality

**Figure 7:**
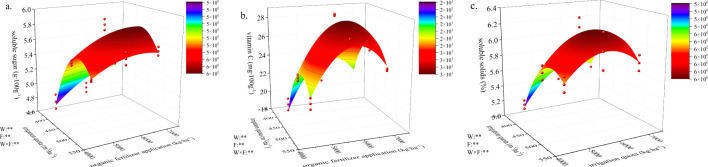
The regression relationship of soluble sugar, vitamin C and soluble solids in pumpkin under water and fertilizer coupling. **(A–C)**, respectively represent the fitted model of soluble sugar, vitamin C and soluble solids in pumpkin, ** represent the significant difference when P is 0.01.

Using average data from 2022 and 2023, along with a two-year data set as an example, regression models were constructed with irrigation quota and organic fertilizer application amount as independent variables, while pumpkin soluble sugar, vitamin C, and soluble solids served as dependent variables (**Figure 7**). Regression analysis showed R² values of 0.920, 0.908, and 0.902 for the models of pumpkin soluble sugar, vitamin C, and soluble solids models, respectively, indicating a good model fit. Furthermore, the models exhibited significance at p<0.05 with F-values of 48.32, 41.61, and 38.56, respectively.

Irrigation, fertilization, and the coupling of water and fertilizer significantly influenced soluble sugars, vitamin C, and soluble solids (P<0.01). Under W1 conditions, the soluble sugar content increased with increasing organic fertilizer application; however, under W2 conditions, it initially increased before subsequently decreasing as organic fertilizer application rose. Vitamin C content in W2 was 20.7% ~ 24.8%, 5.5% ~ 6.5%, and 28.9% ~ 38.5% higher than those in W1, W3, and CK treatments, respectively. The highest vitamin C content was observed in the W2F2 treatment, while the lowest was found in the W1F1 treatment. Under F2 and F3 conditions, the soluble solids content showed an initial increase followed by a decrease with increasing irrigation amounts. At equivalent irrigation quota, the soluble solids content of the F2 treatment was found to be between 6.5% ~ 7.9%, 0.25% ~ 3%, and 9.4% ~ 10.7% higher than that of the F1, F3 and CK treatments, respectively.

**Figure 8:**
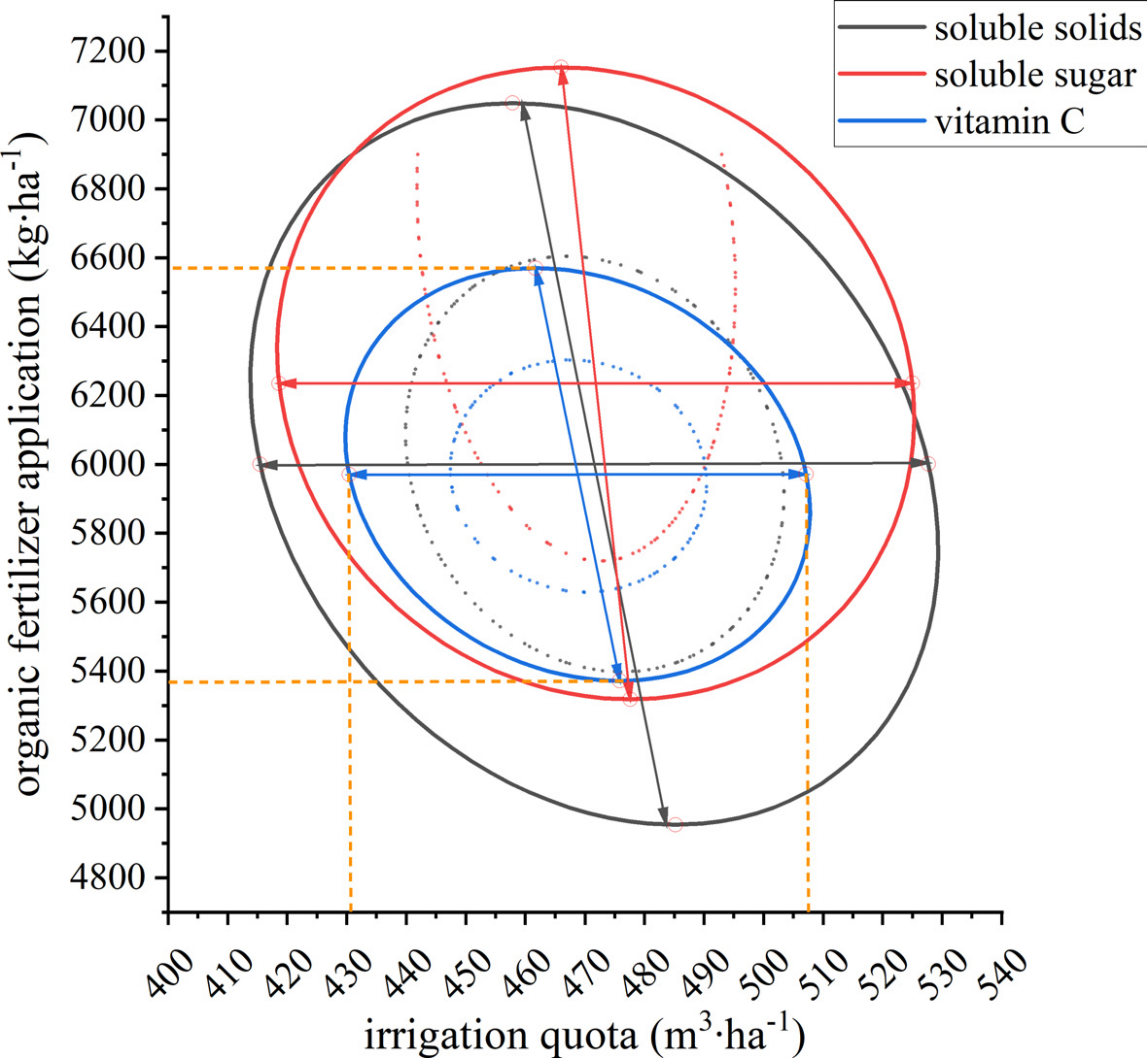
The optimal range of pumpkin quality under the coupling of water and fertilizer. Black ellipses, red ellipses, and blue ellipses indicate 95% optimal confidence intervals for soluble solids, soluble sugar, and vitamin C, respectively. The horizontal coordinates are the range of irrigation quotas covered by the confidence intervals, and the vertical coordinates are the range of organic fertilizer application covered by the confidence intervals.

To comprehensively assess the overall quality of pumpkin, the confidence ellipse was constructed and superimposed with 95% of the three quality indicators reaching the maximum value as the optimization condition (**Figure 8**). The optimal range of water and fertilizer coupling regulation to ensure the optimal quality of pumpkin was obtained: the ideal range of irrigation quota was 430-506 m^3^·ha^-1^, and the optimal range of organic fertilizer application was 5,373-6,570 kg·ha^-1^. In summary, the soluble sugar, vitamin C and soluble solids of the W2F2 treatment were all higher, and the pumpkin quality was the best.

### 3.6 Comprehensive evaluation

Six evaluation indices across three dimensions — yield, water and fertilizer use efficiency (IWUE, PFP), and quality (soluble sugar, vitamin C, soluble solids) — were selected for the comprehensive evaluation using the TOPSIS method. The relative fitness and overall rankings of different combinations of irrigation quotas and organic fertilizer applications were presented in **Table 3**. In both 2022 and 2023, the W3F2 treatment achieved the highest overall ranking, followed by the W2F2 treatment. Conversely, the W1F1 treatment had the lowest ranking. Compared to the W1 treatment, both the W2 and W3 treatments demonstrated higher relative fitness and overall rankings. Additionally, the F2 treatment (excluding W1F2) exhibited superior relative fitness and overall rankings compared to the F1 and F3 treatments.

**Table 3:** Relative fit and comprehensive ranking based on TOPSIS comprehensive evaluation method.

Treatment	2022		2023	
	Relative fitness	Ranking	Relative fitness	Ranking
W1F1	0.163	10	0.117	10
W1F2	0.301	9	0.306	9
W1F3	0.351	6	0.357	6
W2F1	0.325	7	0.31	8
W2F2	0.536	2	0.547	2
W2F3	0.444	4	0.449	4
W3F1	0.304	8	0.336	7
W3F2	0.554	1	0.561	1
W3F3	0.388	5	0.366	5
CK	0.474	3	0.49	3

## 4 Discussion

### 4.1 Impact on soil moisture

Soil moisture was a critical component of the soil and served as a vital source for water uptake by crops (Farrell et al., 2023). The status of soil moisture reflected the ability of soil to supply water, significantly influencing crop growth and yield. Results from Wang et al. (2024) indicated that the substitution of organic fertilizer for chemical nitrogen fertilizer reduced soil bulk density and solid phase, while increasing total soil porosity and water content. Furthermore, the application of organic fertilizer was shown to mitigate drought conditions (Tripathi et al., 2014). The field experiment conducted in this study demonstrated that the application of organic fertilizer significantly increased soil moisture content within the 0 ~ 50 cm soil layer. This enhancement was attributed to the optimization of soil structure facilitated by organic fertilizers, which increased both soil porosity and organic matter, thereby substantially improving soil water retention. During the decomposition of organic fertilizers, the formation of soil aggregates was promoted, further enhancing both water retention and permeability (Bai et al., 2024). Concurrently, organic fertilizers stimulated microbial activity in the soil, which not only promoted the development and stability of soil structure, but also indirectly enhanced its water-holding capacity (Jin et al., 2023), creating favorable conditions for both soil health and crop growth.

### 4.2 Effects on pumpkin growth

The judicious regulation of water and fertilizer application was a critical factor in promoting crop growth. Mu et al. (2023) demonstrated that irrigation and fertilization significantly affected the growth of Panax notoginseng. This study revealed that the regulation of water and fertilizer had a significant effect on both stem length and diameter of pumpkin (p < 0.05), with adequate irrigation combined with organic fertilizer application enhancing both stem diameter and length. Excessive fertilization could lead to water deficiency. Wang et al. (2023) demonstrated that the application of organic fertilizer under deficit irrigation could enhance dry matter accumulation in maize in semi-arid regions. This study found that at the same level of fertilization, dry matter accumulation in pumpkin was greater under high irrigation quota compared to medium and low irrigation quota, aligning with the findings of Guo et al. (2022). Under specific irrigation conditions, the F2 fertilization treatment exhibited superior dry matter accumulation in all growth periods, indicating that a moderate level of fertilization optimizes pumpkin growth and promotes efficient dry matter accumulation.

### 4.3 Effects on pumpkin yield, water and fertilizer use efficiency

Pumpkin yield increased with higher irrigation quota, which was associated with an enhanced soil water availability. Conversely, the relationship between organic fertilizer amount applied and pumpkin yield exhibited an initial increasing trend, followed by a decreasing trend, indicating the existence of an optimal fertilization level. Exceeding this threshold may result in diminishing marginal returns. This study demonstrated that excessive fertilization could lead to reduced yields, with the W3F2 treatment producing the highest yield — 26% greater than the control (CK) treatment — suggesting that a judicious combination of water and fertilizer could effectively enhance pumpkin yield. Yan et al. (2022) demonstrated that excessive irrigation and fertilization not only led to resource wastage and environmental pollution, but also did not necessarily ensure high wheat yields due to plant overgrowth. Zhang et al. (2023a) discovered that moderate deficit irrigation can enhance crop yield, whereas excessive irrigation impairs root soil aeration and affects yield. In our study, the high irrigation quota (W3) increased pumpkin yield by 7.91% compared to the medium quota (W2), likely due to climatic differences, especially 363 mm more annual precipitation at Zhang’s site. These findings underscore the importance of tailored irrigation strategies for optimizing crop production under specific environmental conditions. In this study, IWUE and PFP, key indicators for evaluating the coupling efficiency of water and fertilizer, exhibited variations with different levels of irrigation and fertilization. Specifically, IWUE gradually decreased as irrigation amounts increased, while PFP showed an increase with higher irrigation levels, this is consistent with the findings of Zhang et al. (2023a). This indicated the need to focus on water and fertilize use efficiency, alongside yield enhancement to achieve sustainable development. Optimizing water and fertilizer management strategies, such as adjusting irrigation quotas and fertilizer applications, may enhance resource use efficiency while minimizing waste.

### 4.4 Effect on pumpkin quality

The pumpkin quality directly affected their flavor, taste and nutritional value, which determined their market value. Vitamin C was an important vitamin in the human diet nutrition that significantly impacted the edible value of pumpkin (Buzigi et al., 2022). It was indicated that a moderate water deficit positively affected the soluble sugar content of pumpkin (Li et al., 2022). Zhang et al. (2024) found that the application of a certain amount of organic fertilizer could significantly enhance both vitamin and soluble sugar content in pumpkin compared to the application of chemical fertilizer alone. Notably, vitamin C content was the highest in the 100% organic fertilizer treatment at 39.20 mg·100g^-1^. In this study, the vitamin C content was the highest in the W2F2 treatment, averaging 28.85 mg·100g^-1^ over two years. The soluble sugar content under the W2 level was 2.74% ~ 3.39%, higher than that under the W3 treatment. In summary, these findings indicated that the application of a certain amount of organic fertilizer and irrigation could enhance pumpkin quality under the experimental conditions, which aligned with the results reported by Youssef et al. (2021).

Although the present study explored the relationship between soil water content, pumpkin growth indicators, yield and quality in response to water-fertilizer coupling, it did not involve the quantitative analysis of the causal relationship between the indicators, and future studies can further explore the intrinsic mechanism of the indicators using regression analysis methods such as structural equation modelling.

## 5 Conclusions

Based on a two-year field experiment, this study elucidated the effects of water and fertilizer coupling on pumpkin growth and quality under organic fertilization conditions.

1. Moderate irrigation combined with organic fertilizer application significantly enhanced soil moisture content especially at 50 cm depth, and significantly increased the dry matter accumulation of pumpkin, with the accumulation rate peaking at 60-80 days after sowing.
2. When only quality indicators were considered, the W2F2 treatment performed well in increasing the soluble sugar, vitamin C, and soluble solids content of pumpkin, thereby optimizing its quality.
3. The W3F2 treatment (525 m³·ha⁻¹ irrigation quota and 5,700 kg·ha⁻¹ organic fertilizer application rate) was a more suitable water-fertilizer coupling management strategy for pumpkin cultivation in the arid region of northwest China than the other treatments when evaluated comprehensively in terms of yield, quality and water and fertilizer use efficiency using the TOPSIS method.

## Data Availability

The raw data supporting the conclusions of this article will be made available by the authors, without undue reservation.
